# Effect of perioperative intravenous lidocaine on the incidence of short‐term cognitive function after noncardiac surgery: A meta‐analysis based on randomized controlled trials

**DOI:** 10.1002/brb3.1875

**Published:** 2020-10-12

**Authors:** Xiao Huang, Yuan Sun, Dandan Lin, Changwei Wei, Anshi Wu

**Affiliations:** ^1^ Anesthesia Department of Beijing Chao‐Yang Hospital Beijing China; ^2^ Pharmacy Department of Beijing Chao‐Yang Hospital Beijing China

**Keywords:** lidocaine, noncardiac surgery, postoperative cognitive dysfunction

## Abstract

**Objectives:**

Postoperative cognitive dysfunction is a debilitating postoperative complication. The perioperative neuroprotective effect of lidocaine has conflicting results.

**Methods:**

In this qualitative review of randomized controlled clinical trials on the perioperative use of lidocaine, we report the effects of intravenous lidocaine on brain function after noncardiac surgery. Studies were identified from PubMed, MEDLINE, and Cochrane Central Register.

**Results:**

Of the 453 retrieved studies, 4 randomized trials were included. No significant association between the use of lidocaine postoperative cognitive states was found (risk ratio 0.67; 95% CI −0.02 to 1.36; I^2^89%; *p* = .06).

**Conclusions:**

Current evidence cannot suggest that perioperative intravenous use of lidocaine has pharmacological brain neuroprotection after noncardiac surgery. All the included studies were small‐scale research, and the total number of participants was small; the results should be interpreted with caution.

## Graphical abstract

Current evidence cannot suggest that perioperative use of lidocaine has pharmacological brain neuroprotection after noncardiac surgery. All the included studies were small‐scale research, and the total number of participants was small; the results should be interpreted with caution.

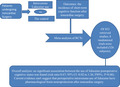

## 1 INTRODUCTION

Postoperative cognitive dysfunction (POCD) is a debilitating surgical complication (Smith et al., 2018), characterized by symptoms such as memory deterioration, loss of concentration, attention‐deficit disorder, mental disorders, and even personality change (Newman et al., 2001). Studies showed that POCD can occur after all types of surgery, not just after heart surgery (Edipoglu & Celik, 2019; Holmgaard et al., 2019; Kristek et al., 2019). The reported incidence of POCD is 26% within a few weeks which decreased to 10% 3 months after noncardiac surgery (Brown & Purdon, 2013). The growing number of publications concerning postoperative cognitive decline (POCD) after noncardiac surgery is indicative of the health and economic issues. Furthermore, we revealed that defining POCD is a very controversial matter. Although there is a lot of research on the pathogenesis and preventive measures of POCD, the incidence of POCD is still stable and has become a common postoperative complication (Li et al., 2017; Luo et al., 2019).

Lidocaine is a common antiarrhythmic agent and widely used in both local and general anesthesia patients (Oni et al., 2012; Weibel et al., 2016). It is a relatively safe compound and makes the surface charge of biological membranes more positive and alters the permeability of the blood–brain barrier (Santa‐Maria et al., 2019). Peripheral and systemic administration of lidocaine was found to reduce brain acetylcholinesterase activity (Abreu et al., 2019). Intravenous lidocaine significantly improves the recovery of neurological function after acute cerebral ischemia (Evans et al., 1984). The extensive research in animal experiments suggests that the conventional dose of lidocaine exhibited a neuroprotective effect (Lin et al., 2012; Popp et al., 2011). However, the mechanisms underlying lidocaine treatment‐induced neuroprotection remain highly controversial.

Results of a meta‐analysis showed that a higher concentration of lidocaine can be an effective neuroprotective agent on patients following heart surgery (Habibi et al., 2018). However, there is an ongoing debate in the literature on the effect of lidocaine on cognitive function in noncardiac surgery (Chen et al., 2015; Peng et al., 2016). They have raised concerns about the effects of lidocaine on postoperative cognition status. It is unclear whether it has neuroprotection in patients following noncardiac surgery. There was no retrospective study focus on the effect of perioperative lidocaine use on cognitive performance after noncardiac surgery. Our group sought to conduct a meta‐analysis of the available literature to clarify the effect of using lidocaine on postoperative cognitive function.

## 2 MATERIAL AND METHODS

### 2.1 Study selection and eligible criteria

We conducted a systematic review of the literature in three main electronic databases (MEDLINE, EMBASE, and the Cochrane databases) to identify all articles up to January 2020 regarding the effect of intravenous lidocaine on the cognitive function which measured by the neuropsychological test. The electronic search strategy used the prespecified keywords and MeSH terms (“AND” or “OR”) to identify the articles of interest. The search terms included the following: lidocaine, xylocaine, “lignocaine,” “lidocaine hydrochloride, POCD, postoperative neurocognitive disorder (PND), delirium, cognitive impairment, cognitive dysfunction, cognitive deficit, neurological complication, cognitive disorders, cognition, cognitive function, noncardiac surgery, surgery. We reviewed the reference lists of all included papers to ensure the inclusion of relevant studies not included in our initial literature search. Only published randomized controlled trials (RCTs) on humans comparing the use of the lidocaine with placebo in adults undergoing noncardiac surgery were considered eligible. We identified RCTs that met the following criteria: (1) used lidocaine with placebo; (2) evaluated neurological status before the operation and within one week after the operation, and measured the cognitive status preoperatively and postoperatively using the same tests; (3) included adult patients (at least 18 yr of age with no upper limit) undergoing noncardiac surgery. Trials were included if data on the above outcomes were available either in the publication or from the author in correspondence. The title and abstract of each citation were independently screened by two sets of two reviewers to identify potentially eligible trials. Two authors (Xiao Huang and Yuan Sun) independently screened and assessed titles, abstracts, and full‐text papers. Disparities were resolved by consensus. If either reviewer felt that the study may contain a relevant trial, the article was retrieved with a full‐text assessment. We attempted to contact the authors of the included articles if further data were required.

Details of the study population, interventions, and outcomes were extracted using a standardized data extraction form that includes eligibility and exclusion criteria, randomization, allocation concealment, blinding, number and characteristics of patients, type of surgery, duration of follow‐up, drug dosage and method of administration, and neuropsychological test. The quality of each randomized trial was assessed using the Cochrane Collaboration risk of bias tool assessing random sequence generation, allocation concealment, blinding process, incomplete outcome data, selective reporting, and other bias (Higgins et al., 2011). If possible, though, we wanted to conduct subanalyses for the underlying disease and the type of surgery. Retrospective studies, registry or chart reviews, and studies without the aforementioned comparators were also to be excluded.

### 2.2 Data extraction

Also, the heterogeneity was measured by the I^2^ which describes the percentage of total variation across studies that is due to heterogeneity rather than chance. A value of 0% indicates no observed heterogeneity, and larger values show increasing heterogeneity (Higgins et al., 2003). When heterogeneity was found we tried to identify and describe the reason. Parametric variables were presented as the mean ± *SD* and nonparametric variables were presented as the median (interquartile range). If continuous data needed to be analyzed, standardized mean differences were to be calculated. All analyses were conducted with Review Manager (RevMan) software version 5.3 (Cochrane Collaboration, Copenhagen, Denmark). For all analyses, a 2‐sided P 0.05 was considered significant. High heterogeneity was considered present with chi‐square test *p* < .10 and/ or I^2^ ≥ 50%. Study heterogeneity was judged to be low based on visual inspection of the forest plot and I^2^ statistic. Considering the possibility of high heterogeneity caused by different study designs, different assessments of POCD, and different administration of lidocaine use, a random‐effect model was used in the present study. All *P* values were two‐tailed with the statistical significance set at < 0.05.

## 3 RESULTS

### 3.1 Eligible studies

**FIGURE 1:**
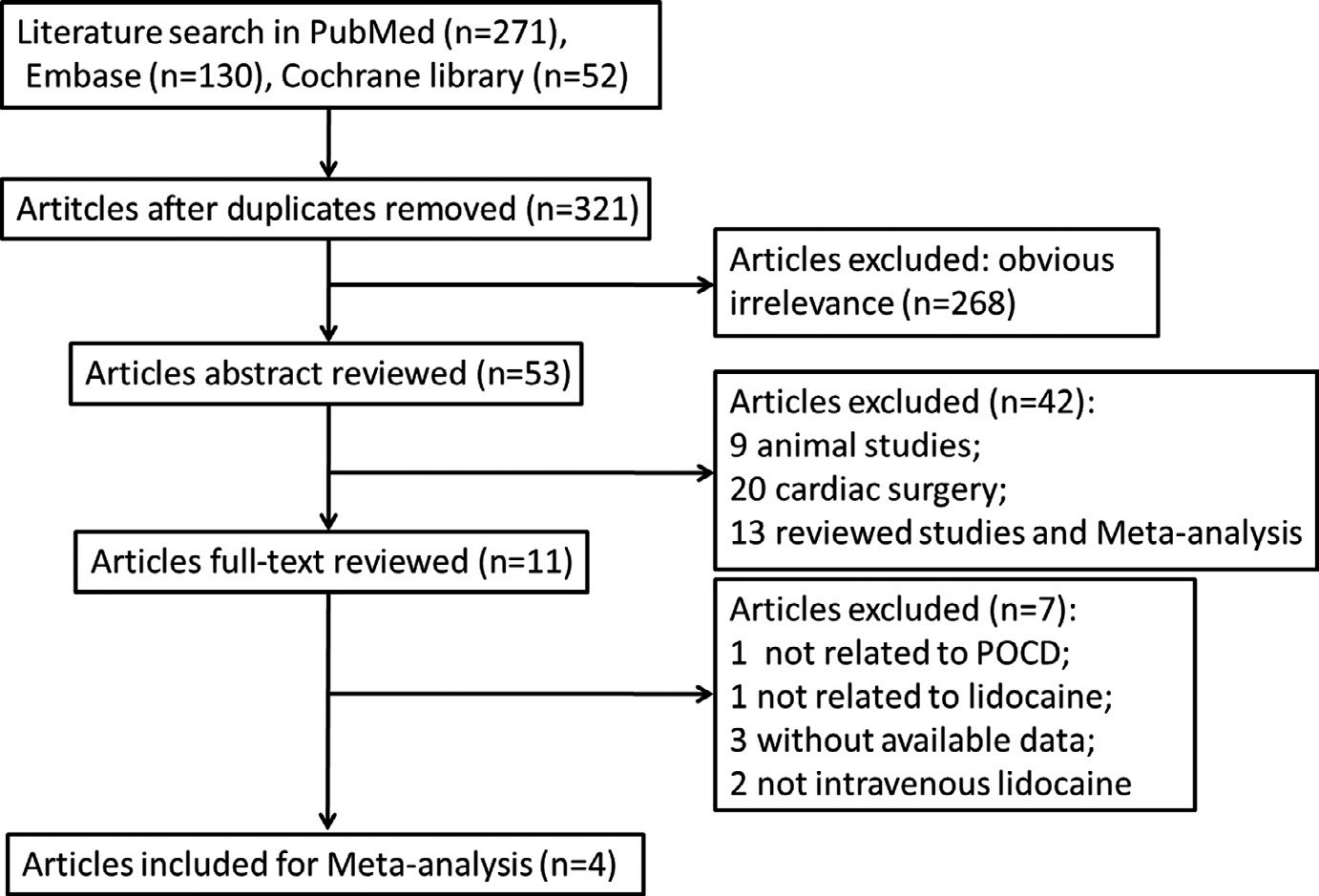
Flowchart of the literature search

The flowchart showing the retrieved results and the process of study selection is displayed in Figure 1. According to the predetermined strategies, 271, 130, and 52 relevant studies were selected from PubMed, EMBASE, and Cochrane Library databases, respectively. After removing the repeated articles, 321 studies remained. A total of 268 ineligible studies were eliminated after browsing tittle. Then, 53 studies were further removed through reading the abstract. Furthermore, 11 studies were selected for full‐text evaluation. Finally, a total of 4 eligible randomized controlled trials were included for the present meta‐analysis (Chen et al., 2015; Guo et al., 2019; Hashemi et al., 2013; Peng et al., 2016).

### 3.2 Study characteristics

Table 1 summarizes the characteristics of the selected trials. A total of 326 subjects (including 162 cases in the lidocaine group and 164 cases in the control group) were included in this meta‐analysis. The time for the studies was published from 2013 to 2019 year. Of the 4 included studies, 3 studies were conducted in China and 1 was in Iran. Both studies assessed cognition on the Mini‐Mental State Examination (MMSE) scale, and they compared intravenous lidocaine with saline. Patients enrolled were of both sexes. The trials differ in time and dosing of the lidocaine infusion. The included population was mainly middle‐aged and elderly patients with age ranging from 44 to 72 yr. The included publications differed in the time frames of postoperative evaluation. The follow‐up time ranged from 1 day to 6 months after surgery. All articles assessed cognition before surgery and the first week after surgery. One study with both short‐term and long‐term cognitive outcomes was evaluated.

**TABLE 1:** Characteristics of the studies included in the meta‐analysis

	Chen et al., 2015	Guo et al., 2019	Hashemi et al., 2013	Peng et al., 2016
Country	China	China	Iran	China
Age cases/controls	71.3/71.8	70.96/71.12	66/67	45/44
Female(%) cases/controls	42.5/37.5	63.8/51.7	22.9/20	50/52.5
Number cases/controls	40/40	58/58	35/35	46/48
Type of surgery	Spine surgery	Orthopedic surgery	Urologic or orthopedic surgeries	Supratentorial tumor surgery
Follow‐up: time and number of patients, cases/controls	Before operation and 3d after surgery.	Before operation and 3d after surgery.	Before operation, discharge from recovery, 6 hr after surgery and 24 hr after surgery.	Before surgery, 24 hr, 1 week, 1 month, 3 months, and 6 months after surgery.
Trial medication administration cases/controls	A bolus of 1 mg/kg of lidocaine over 5 min administered after induction of anesthesia and followed by a continuous infusion at 1.5 mg kg^−1^ h^−1^ until the end of the surgery; normal saline.	A bolus of 1 mg/kg of lidocaine in 5 min after induction of anesthesia and then a continuous infusion at 1.5 mg kg^−1^ h^−1^ until the end of the surgery; normal saline.	Intravenous lidocaine (1.5 mg/kg) administered 1 to 2 min before extubation; normal saline.	Administered as an intravenous bolus (1.5 mg/kg) after anesthesia induction followed immediately by infusion at 2 mg kg^−1^ h^−1^ in a normal saline vehicle until the end of surgery; normal saline group.
Neuropsychological test	MMSE	MMSE	MMSE	The MMSE and the Information–Memory–Concentration test (IMCT), and the Hamilton Rating Scale for Depression (HRSD) and the Hamilton Rating Scale for Anxiety (HAMA).

Abbreviation: MMSE, Mini‐Mental State Examination.

### 3.3 Risk of bias

**FIGURE 2:**
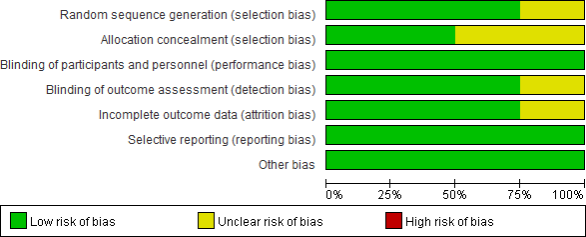
Bias risk of the eligible studies

**FIGURE 3:**
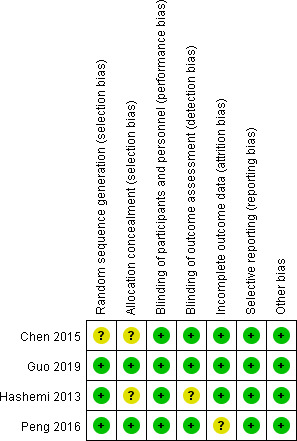
Sensitivity and specificity of the included studies. “+” represents low risk of bias; “?” stands for unclear risk of bias

The risk of bias graph and summary for the individual studies is reported in Figures 2 and 3, and the methodological bias of the included studies was relatively low, indicating the high qualities of the eligible studies. We analyzed the relationship between lidocaine use and POCD in 4 RCTs. Among 4 RCTs, 1 RCT did not report the method of random sequence generation, 2 RCTs did not report the method of allocation concealment and had unclear risk of bias, 1 trial demonstrated unclear risk of bias in blinding of outcome assessment, and 1 study was considered to have unclear risk of bias in the incomplete outcome.

### 3.4 Meta‐analysis

**FIGURE 4:**
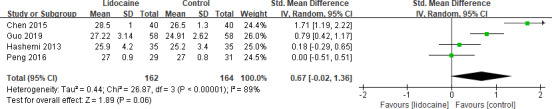
Comparison of postoperative cognition between the lidocaine group and the control group

All the articles showed no significant differences in preoperative tests, and the score decreased after surgery with a cognitive decline of varying degrees. Lidocaine cannot reduce the incidence of POCD (risk ratio 0.67; 95% CI −0.02 to 1.36; I^2^89%; *p* = .06). A meta‐analysis with a random‐effects model showed a similar risk of postoperative cognitive decline with or without the use of lidocaine (Figure 4).

## 4 DISCUSSION

POCD is a common postoperative complication and lacks effective prevention measures (Carr et al., 2018). Many pharmacological neuroprotective applications have been documented, but all were controversial (Li et al., 2019; Rasmussen et al., 2016). To explore whether lidocaine was neuroprotective or neurotoxic, the effect of lidocaine on postoperative cognitive function was fully analyzed in the current meta‐analysis. According to the predetermined criteria, a total of 4 studies were eligible and thus were included for this meta‐analysis. Lidocaine was used in our analysis and cognition was the independent end point. we did not find the neuroprotective effect of intravenous lidocaine on noncardiac surgery. Combined analyses inferred that there was no significant association between lidocaine and POCD. All the included studies were small‐scale research and the total number of participants was small; the results should be interpreted with caution. And we realized that a high heterogeneity existed in our study. It is necessary to carry on subanalysis by surgical classification. We did not conduct a subgroup analysis based on surgery for lacking adequate inclusion studies.

Chen et al. concluded that lidocaine may be an effective neuroprotective agent in treating early postoperative cognitive dysfunction in elderly patients undergoing spine surgery (Chen et al., 2015). Guo et al. found that lidocaine had neuroprotective effects on early POCD in elderly patients undergoing orthopedic surgery and may be associated with decreased cerebral oxygen and anaerobic metabolism (Guo et al., 2019). Hashemi et al. demonstrated that intravenous lidocaine administration at the end of surgery and before extubation had no prominent effect on the improvement of cognition impairments in the elderly undergoing noncardiac surgery with general anesthesia (Hashemi et al., 2013). Peng et al. found that intraoperative infusion of lidocaine does not significantly decrease the incidence of postoperative neuropsychological‐cognitive decline in patients 6 months after supratentorial tumor surgery (Peng et al., 2016). Moreover, no significant publication bias was found for POCD. These conflicting results in the 4 eligible studies might result from different sample sizes and lidocaine administrations. Therefore, it was necessary to conduct the present meta‐analysis for assessing the diversity of these studies using a quantitative evaluation approach.

Current evidence suggests that pharmacological brain neuroprotection might decrease the incidence of postoperative neurological deficits (Bilotta et al., 2013). The aim of this review of RCTs on perioperative pharmacological brain neuroprotection was to evaluate the effects of lidocaine on postoperative cognitive function. In the present meta‐analysis, the cognitive states after surgery did not differ between treated patients and the control group. Our analyses indicate an obvious lack of neuroprotective effects of intravenous lidocaine on the cognition following noncardiac surgery. Our results differ from previous studies on the brain‐protective mechanisms of lidocaine. Lidocaine attenuated the production of proinflammatory cytokines and has anti‐inflammatory effects and potential as an anti‐inflammatory agent (Caracas et al., 2009; Su et al., 2010). Moreover, lidocaine can reduce hippocampal neuronal death and inflammatory events (Chiu et al., 2016). Neuroinflammaging makes a huge difference in the mechanism of POCD (Safavynia & Goldstein, 2019). Lidocaine can reduce neuronal cell death in rat hippocampal slice cultures (Cao et al., 2005). Coadministration of lidocaine and dexmedetomidine improves the neurological outcome in rats (Goyagi et al., 2009). But in this study, there is yet sufficient evidence to prove a suggestion for routine use of lidocaine in cardiac surgery for neuroprotection. At the same time, the comprehensive neuropsychological assessment requires a battery of tests assessing a battery of cognitive domains. However, there is no consensus for detecting and quantifying neurological impairment and POCD. A widely accepted POCD definition has not been established, and the pathogenesis is still unclear (Needham et al., 2017; Wang et al., 2014). Best practices for postoperative evaluation of POCD have also not been determined. In this review, we included only articles that used consistent pre‐ and postoperative evaluation methods. Underestimateing the importance of POCD can cause significant health‐related and economic‐related completions (Roach et al., 1996). Future investigations must provide more insights into these issues.

In this study, the meta‐analysis was firstly used for evaluating the correlations between intravenous lidocaine and POCD following noncardiac surgery. Our study has many strengths. We only included articles on intravenous lidocaine, reducing the differences caused by different applications such as topical anesthesia or infiltration anesthesia. At the same time, POCD was evaluated within one week after surgery in the included studies to explore the short‐term effects of lidocaine on POCD. POCD is characterized by cognitive impairment from 3 days to 1 year or even several years after surgery. The present study reduces variation between assessments at different time points. Limiting the review to RCTs that compared lidocaine with placebo, we were able to determine the relative risk of cognition impairment and minimize cohort studies or retrospective studies bias.

We notice there are several limitations in this meta‐analysis. First, heterogeneity among the studies should not be overlooked. Heterogeneity was relatively high, which might be clinical and methodological heterogeneities induced by inconsistent operation types, included population, lidocaine administration, and grouping standards. Moreover, the number of eligible studies was relatively small. Trials included were limited to a few, small, single‐center studies, only six studies included in our meta‐analysis. And the few numbers of total participants make this meta‐analysis underpowered for the outcomes. Thereby, the evidence of a neuroprotection role of lidocaine is not conclusive yet. And publication bias would be difficult to evaluate. The diagnosis of POCD mainly depends on neuropsychological scales to assess global cognitive status, short‐term and intermediate‐term memory, attention, concentration, and psychomotor skills. The main tool used in the included studies was MMSE, which may be inadequate in assessing executive function. A study assessing patients undergoing intracranial surgery was included, and this population is extremely different and particularly susceptible to cognitive changes. Last but not most, there were variations in lidocaine dosage and timing of prescription. Future studies need to include a broader range of relevant clinical scenarios using a wider consensus on the methodological approaches, including timing and dosing of drug administration, patient selection, and perioperative neurological and cognitive testing. Considering the limitations of our study, it is urgent to design high relevant large clinical trials in the future.

## 5 CONCLUSIONS

In the present meta‐analysis, we analyzed the literature on the potential neuroprotective effects of intravenous lidocaine. Reliable clinical trials on this neuroprotection are still rare. Our assessment based on 4 surgical studies showed that there are currently insufficient data to show protection by lidocaine against postoperative cognitive dysfunction following noncardiac surgery. More effective and safe, therapeutic interventions are urgently needed.

## CONFLICT OF INTEREST

The authors declare that there is no conflict of interest. This study received no specific external funding.

## AUTHOR CONTRIBUTIONS

Anshi Wu and Changwei Wei conceived and designed the study. Xiao Huang, Yuan Sun, and Dandan Lin collected and assembled the data. Yuan Sun and Xiao Huang analyzed the data. All authors wrote the first draft of the manuscript and taken part in the final approval of the manuscript.

## ETHICAL STATEMENT

The present study does not require an ethical statement.

## Data Availability

Data available.
